# *Strongylodon juangonzalezii*, a remarkable new species of *Strongylodon* (Fabaceae) from Mulanay, Quezon Province, Philippines

**DOI:** 10.3897/phytokeys.73.10055

**Published:** 2016-10-18

**Authors:** Annalee S. Hadsall, Michelle DR. Alejado, Ariel R. Larona, Ivy Amor F. Lambio

**Affiliations:** 1 Institute of Biological Sciences, College of Arts and Sciences, University of the Philippines Los Baños, 4031 Laguna, Philippines; 2 Museum of Natural History, University of the Philippines Los Baños, 4031 Laguna, Philippines

**Keywords:** Mulanay, Fabaceae, Quezon, Philippines, Strongylodon

## Abstract

A new species, *Strongylodon juangonzalezii* Hadsall, Alejado & Cajano, collected from Buenavista Protected Landscape, Mulanay, Quezon, is hereby described. The new species is remarkable for its plagiotropic dense inflorescence made up of 27–31 flowers per cluster in a lateral branch. Flowers are lilac when young, then gradually turn blue when mature. A comparison of the morphology of *Strongylodon juangonzalezii* and related species of *Strongylodon* in the Philippines is provided. Detailed illustration based on the holotype and photos from its natural habitat are also included. With this new species, the Philippines now harbors eight endemic species of *Strongylodon*. A key to distinguish the species is provided.

## Introduction

*Strongylodon* (Fabaceae – Papilionoideae – Erythrininae) was described as a genus in 1836 by Julius Rudolph Theodor Vogel. Its distribution includes Madagascar and Reunion to Sri Lanka, India, Australia, and northward to the islands of the Pacific (Polhill 1912, Huang 1991, Tropicos 2016). In the Philippines, *Strongylodon* is currently distributed in the islands of Luzon (Abra, Cagayan, Bataan, Rizal, Cavite, Laguna, Quezon, Sorsogon, Catanduanes, Mindoro, Aurora Province, Benguet, Ilocos Norte, Isabela, Camarines Sur), Visayas (Biliran, Panay) and Mindanao (Agusan del Norte, Zamboanga, Davao, Lanao, Bukidnon) (Merrill 1923, Pelser et al. 2011).

The genus derived its name from the Greek words “strongylos” meaning ‘round’ and “odontos” means ‘toothlike’, referring to the rounded teeth of the calyx. It is also known to exhibit inflorescences in drooping racemes whose color ranges from purplish blue to bluish green to red or orange red (Huang 1991, Takedaa 2010).

Merrill (1923) enumerated 10 species of *Strongylodon* in the Philippines, of which 9 are endemic [*Strongylodon agusanensis* Elm., *Strongylodon caeruleus* Merr., *Strongylodon crassifolius* Perk., *Strongylodon elmeri* Merr., *Strongylodon macrobotrys* A. Gray, *Strongylodon megaphyllus* Merr., *Strongylodon paucinervis* Merr., *Strongylodon pulcher*, *Strongylodon zschokkei* Elm.] with one indigenous (*Strongylodon lucidus* (Forst.f.) Seem.). Huang (1991) revised the entire genus resulting in eight species for the Philippines, including a new species, *Strongylodon loheri* and three synonymized species (*Strongylodon agusanensis* a synonym of *Strongylodon pulcher*, *Strongylodon megaphyllus* a synonym of *Strongylodon macrobotrys*, and *Strongylodon paucinervis* a synonym of *Strongylodon caeruleus*). Currently there are seven species, with *Strongylodon crassifolius* reported as insufficiently known (Pelser et al. 2011). The Plant List, an online database (http://www.plantlist.org, 2013), presently recognized 14 species of *Strongylodon*.

Four sections comprise the genus, namely: Strongylodon, Archboldianus, Macrobotrys, and Craveniae (Huang 1991). All Philippine species of *Strongylodon* belong to section Macrobotrys characterized by having peltate stipules, brachyblast with more than three-flowers, and purplish blue or bluish-green inflorescences.

**Figure 1.**
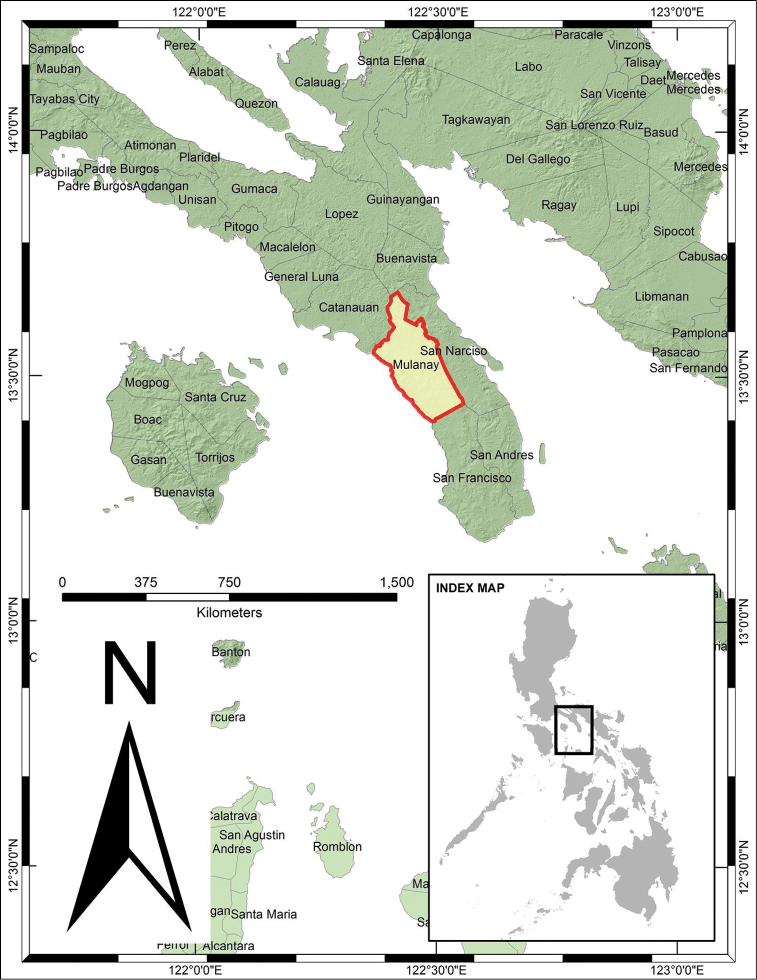
Map of Luzon Island showing the geographic location of Mulanay, Quezon Province, Philippines.

In February 2015, a collaborative field study between University of the Philippines Los Baños-Museum of Natural History
(UPLB-MNH) and local government unit (LGU) of Mulanay resulted in the collection of 128 plant species belonging to 49 families and 90 genera, with most of the species endemic to the Philippines (Fig. 1). An interesting result of the field study was the discovery of a unique specimen of *Strongylodon*. Morphological traits were not consistent with other species within the genus. Although similar to *Strongylodon caeruleus*, novel traits include plagiotropic dense inflorescence and young flowers that are lilac-colored then gradually turning blue when mature.

## Species treatment

### Strongylodon juangonzalezii

Taxon classificationPlantaeFabalesFabaceae

Hadsall, Alejado & Cajano sp. nov.

urn:lsid:ipni.org:names:77158185-1

Figures 2 , 3 , 4

#### Diagnosis.

*Strongylodon juangonzalezii* a habens inflorescentiae racemi spicae densi plagiotropici, lilacinus cum iuvenibus et caerulei cum maturibus, et cum brachyblastae cylindricae et magis quam tres flores in congeners differt.

*Strongylodon juangonzalezii* differs from other species of *Strongylodon* in having dense plagiotropic raceme inflorescence with flowers that are lilac when young and turn blue when mature, and with brachyblasts that are cylindrical and more than 3 flowered.

**Figure 2.**
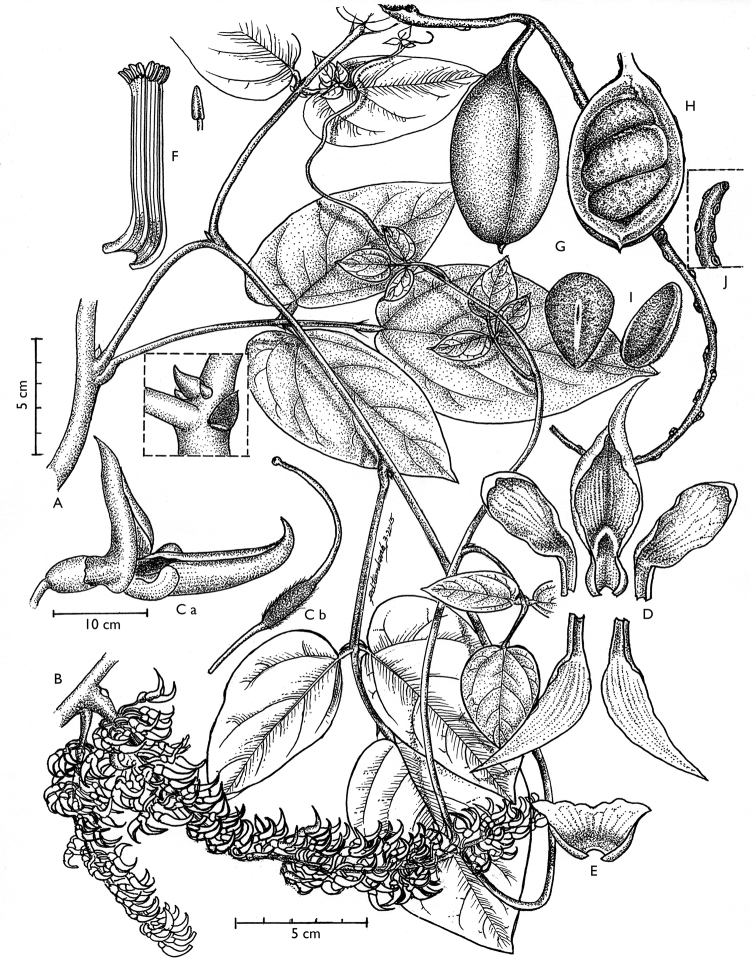
*Strongylodon juangonzalezii* sp. nov. **A** growth habit, inset shows the distinct middle and lateral stipules **B** portion of a flowering branch **C** Detached flower **D** dissected flower **E** calyx **F** androecium and anther **G** intact pod **H** pod opened to show the seeds **I** seeds, front and side view **J** brachyblast.

**Figure 3.**
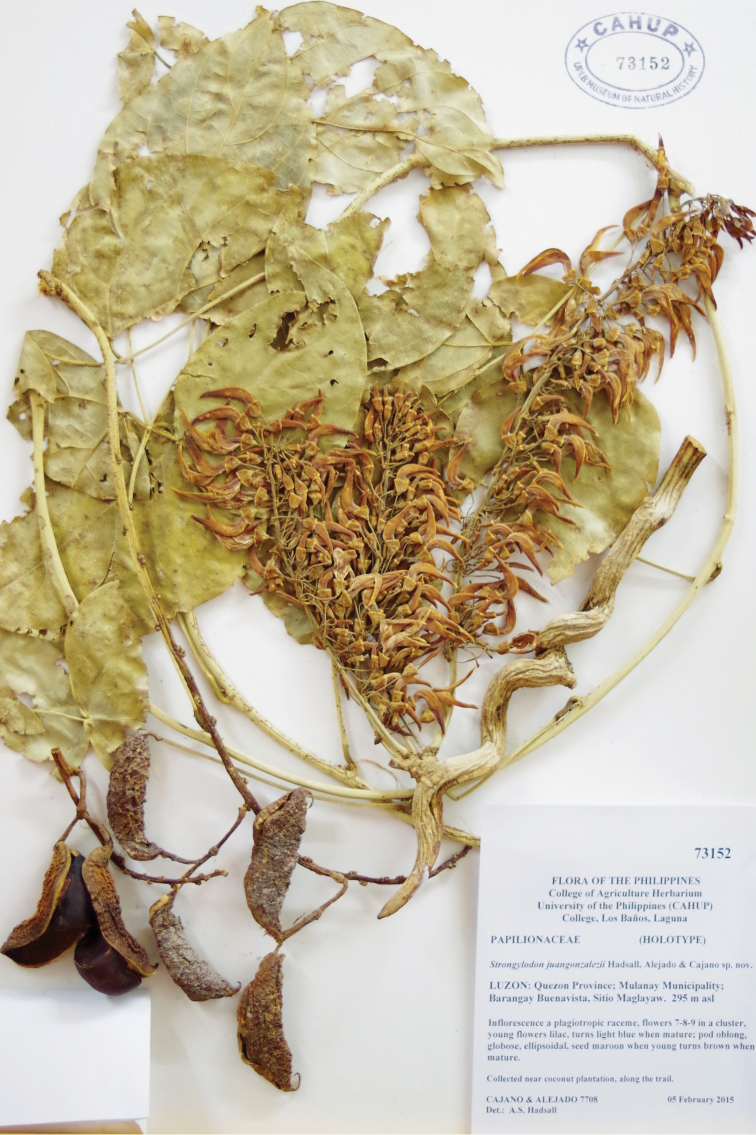
Voucher specimen of *Strongylodon juangonzalezii* sp. nov.

**Figure 4.**
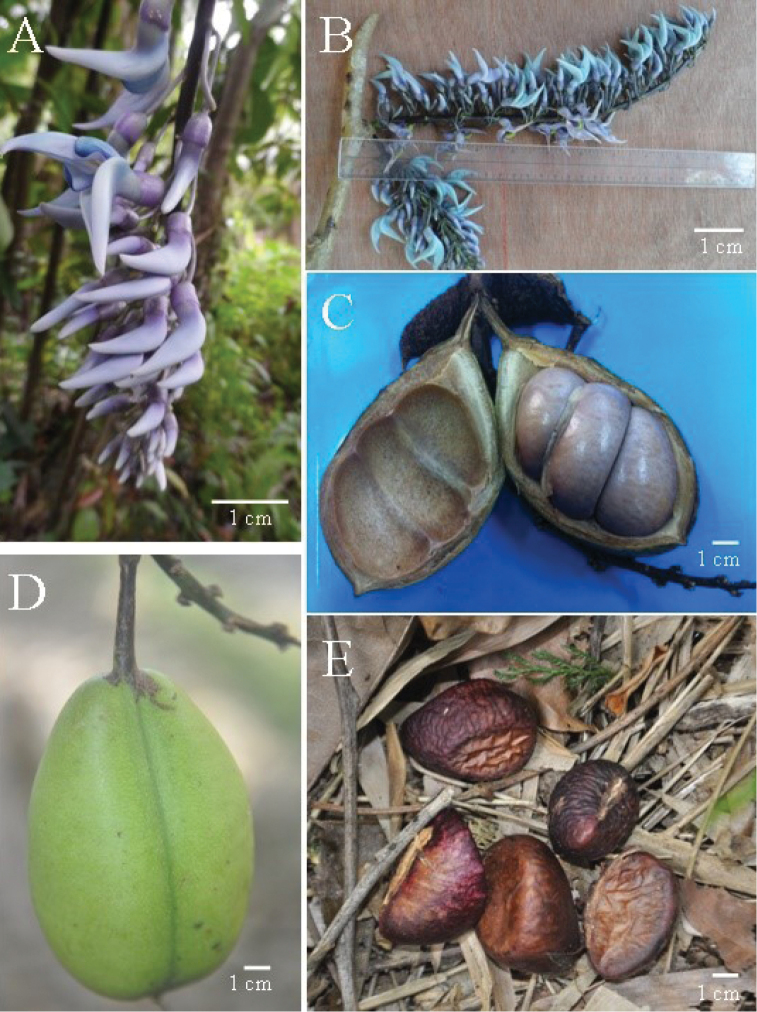
*Strongylodon juangonzalezii* sp. nov. **A** inflorescence **B** inflorescence showing point of attachment **C** opened pod to show seeds **D** young pod **E** mature seeds from the wild. Photographs by Mary Ann O. Cajano (deceased 6 December 2015) and Michelle DR. Alejado.

#### Type.

PHILIPPINES. Luzon, Island, Quezon Province, Municipality of Mulanay, Barangay Buenavista, Sitio Maglayaw, Buenavista Protected Landscape (BPL), 13°31'20"N, 121°24'15"E, 295 m, 5 February 2015, *Cajano & Alejado 7708* (holotype CAHUP 73152!, isotype PNH).

#### Description.

Woody vine reaching the top of the canopy. Mature branches glabrous with lenticels. Leaves 3-foliolate, each 3-nerved, adaxial and abaxial surfaces dark green, margin entire, apex acute, base rounded; lateral leaflets broadly-ovate, oblique, 9 cm long, 6.2 cm wide; terminal leaflet ovate-elliptic, 10.4 cm long, 6.0 cm wide; petiole green, glabrous, base swollen, 12.4–12.6 cm long; rachis green, glabrous, 2.8–2.9 cm long; petiolule green, glabrous, base swollen, 1.2 cm long; stipules three, basifixed, axillary, middle one cylindrical and persistent, lateral ones caducous, leaving conspicuous scars. Inflorescence a dense plagiotropic raceme, up to 12.0 cm long, branches alternating on the main axes; peduncle 2–8 cm, shorter than flowering axis; lateral flowering branches 13.0–27.0 cm long, occurring in pairs, arising from node; pedicel 1.7–1.9 cm long; brachyblasts warty, more than 3-flowered, 5 mm long and 1 mm wide. Flowers 7–9 in a cluster, arranged alternately, 27–31 flowers in a cluster in a lateral branch, with outer flowers opening first; young flowers lilac; calyx lilac, cup-shaped, glabrous, entire; standard petal lanceolate, 2.5–2.6 cm long, 1.3–1.4 cm wide, basal portion ridged, both surfaces of standard petal turns light blue when mature; wings oval 1.2–1.4 cm long, 0.6–0.7 cm wide, slightly auricled at base, upper surface of wings from margin up to 3 mm turns blue when mature, lower surface white; keels lanceolate, 2.8–3.0 cm long, 1.2–1.3 cm wide, both surfaces turn light blue when mature. Ovary pubescent. Pod oblong, globose, unilocular, continuous 6.1–7.5 cm long, 3.9–4.0 cm wide, base rounded, apex aligned with longitudinal axis of fruit, with a green hook, surface glabrous, green with irregular brown marks, dehiscing longitudinally along both sutures. Seeds smooth, symmetrical, 2–3 in a pod, dorsal portion flattened, ventral portion inflated, 2.7–3.1 cm long, 2.1–2.4 cm wide; seed position transverse to fruit length; funiculus whitish, running along ventral side of seed, when mature funiculus is detached leaving a flat scar; hilum linear, around 1.6–1.8 cm of seed, white, with light brown rim; raphe visible; seed coat maroon and shiny when fresh, turning dark brown, papery and wrinkled when mature, not adhering to inner fruit wall.

**Table 1.** Morphological comparison of *Strongylodon juangonzalezii* sp. nov. with other Philippine species of *Strongylodon*.

Characters	*Strongylodon juangonzalezii*	*Strongylodon caeruleus*	*Strongylodon elmeri*	*Strongylodon lucidus*	*Strongylodon macrobotrys*	*Strongylodon pulcher*	*Strongylodon zschokkei*	*Strongylodon loheri*
**Terminal leaflet**	ovate-elliptic	ovate	elliptic to ovate-elliptic	ovate, wide ovate or orbicular	elliptic to ovate-elliptic	ovate-elliptic, elliptic or oblong	elliptic or ovate	ovate
**Size of terminal leaflet (cm)**	10.4 × 6	11–19.5 × 6.5–11	10.5–19 × 3.5–7	0.6–1.3 × 0.55–0.9	12–15.5 × 5.5–7.3	13–22 × 4.2–13	8.5–16 × 3.3–6.7	10–15 × 6–7.5
**Lateral leaflet**	broadly ovate	ovate	ovate	ovate	ovate	oblong	ovate	ovate
**Size of lateral leaflet (cm)**	9 × 6.2	9–16 × 4–8	7.5–16 × 3–7	5–12 × 3–8	9–15 × 3.5–8	11.5–19 × 4.5–10.5	9–13 × 2.8–6	10–13.5 × 4.5–5.5
**Flowering habit**	dense plagiotropic raceme	axillary raceme	compact, subglobose, terminal raceme	pendulous, terminal raceme	pendulous, axillary to terminal raceme	pendulous, terminal raceme	pendulous terminal raceme	pendulous raceme
**Length of inflorescence axis (cm)**	13–27	21–24	18–60	5.5–30	150	3.5–11	19–29	10–19
**Brachyblast**	warty	cylindric	warty	warty	warty	cylindric	warty	warty
**Number of flowers in a cluster**	7–9	7–14	no available data	2–3	5–8	4–10	4–7	5–6
**Color of flowers**	Lilac when young, then blue when mature	purplish-blue	bluish-green	orange-red	bluish-green	purplish-blue	purplish-blue	purplish-blue
**Pedicel length (cm)**	1.7–1.9	1–1.8	3	1.0–2.8	1.8–4	1.5–2.0	1.8–2.3	2.3–2.5
**Calyx**	lilac, cup-shaped	purplish, campanulate	green, campanulate	green, campanulate	purplish, campanulate	green, campanulate	blue, campanulate	green, campanulate
**Standard petal**	lanceolate,	ovate–lanceolate	ovate	lanceolate–ovate	ovate, reflexed	lanceolate	ovate, reflexed	oblong
**Size of standard petal (cm)**	2.5–2.6 × 1.3–1.4	2.6–2.8 × 1.2–1.6	2–3 × 1.1–1.6	1.7–3.1 × 1–1.2	3.7–4.8 × 1.7–2.5	2.1–2.5 × 0.9–1	2.9–3 × 1–1.5	3–3.3 × 1.8–1.9
**Wing petal shape**	oval	oblong	oblong	oblong	oblong–elliptic	oblong	oblong	oblong
**Size of wing petal (mm)**	12–14 × 6–7	12–14 × 5–7.5	11–13 × 5.5–7	7–11 × 3.5–6	20–24 × 8–10	8–11 × 3–4.5	10–13 × 5–6	14–17 × 7–9
**Size of keel petals (mm)**	28–30 × 12–13	27–28 × 4–6	20–28 × 6–8	14–28 × 4–9	45–48 × 11–13	21–23 × 4–5	26–28 × 5–6	29–35 × 7–9.5
**Pod shape**	oblong, globose	elliptic, inflated, rugose	elliptic, rugose	elliptic to elliptic-orbicular	elliptic, inflated, rugose	elliptic, inflated, rugose	elliptic, compressed	oblong or elliptic, inflated
**Size of pod (cm)**	6.1–7.5 × 3.9–4.0	3.5 × 2.1	4–7.5 × 2.2–4	3–8 × 2–4.5	8.5–13 × 6	5.5 × 3.5	9 × 3.5	4.5–6 × 2–2.5
**Altitude (m)**	295	500–1200	low to medium altitude up to 1600	0–1500	110–1000	80–1200	ca. 1400	1300–1900

#### Etymology.

This new species is named after Dr. Juan Carlos Tecson Gonzalez, current director UPLB-MNH, professor of zoology, one of the Philippines ten outstanding young scientists in 2011, a passionate conservationist and ornithologist.

#### Distribution.

So far only two thriving lianas of this species are known from Buenavista Protected Landscape, Mulanay, Quezon Province where it was collected.

#### Habitat and ecology.

This liana thrives in a disturbed secondary growth forest climbing atop a large tree at an altitude of 295 m. The area is adjacent to an old coconut plantation.

#### Phenology.

Flowering and fruiting from February to mid-March.

#### Additional specimens examined.

Other species of *Strongylodon* collected in the Philippines were also examined.

*Strongylodon caeruleus* Merr., Luzon Island, Laguna Province, *ML Steiner 1742*, March 1959, (PNH);

*Strongylodon elmeri* Merr., Luzon Island, Laguna Province, *ML Steiner s.n.*, 17 April 1955, (PNH);

*Strongylodon macrobotrys* A. Gray Exsicc. *Gates CA 1442*, *1443*, *1444*; *Hernaez CA 12426*; *Orlido CA 10250*; *Pancho CA 18190*, *Reyes CA 2921* (CAHP);

*Strongylodon pulcher* C.B. Robinson, Mindanao Island, Agusan Province, *C. Mahesa & J. Escasina s.n.*, 23 February 1967, (PNH); Visayas Island, Leyte Province, *G.E. Edano 14235*, 15 March 1950, (PNH); Mindanao Island, Bukidnon Province, *MD Sulit s.n.*, 10 March 1949, (PNH); Visayas Island, Leyte Province, *G. Edano s.n.*, February 1923, (PNH);

*Strongylodon zschokkei* Elmer, Luzon Island, Mountain Province, *M. Celestino s.n.*, 13 March 1948, (PNH).

#### Conservation status.

All the materials used in this study were collected from a single population known only from the type locality in a region that is still poorly known botanically. This was the first documentation done inside the protected area. We suggest the preliminary conservation status of this species as Data Deficient (DD; IUCN 2014) and endemic to Luzon Island.

#### Discussions.

*Strongylodon juangonzalezii* exhibits plagiotropic branches where the dense racemose inflorescences are attached. In the wild, two colors of the flowers are exhibited – lilac color can be observed in young or newly-opened flowers while the mature ones are blue. This is quite remarkable compared with other species of *Strongylodon* whose flowers retain the same color from bud to fully opened stage. Its pod is oblong and globose while the rest of the species are elliptic. Three shapes of wing petals exist in *Strongylodon*. It is oval in *Strongylodon juangonzalezii*, oblong on *Strongylodon caeruleus*, *Strongylodon elmeri*, *Strongylodon lucidus*, *Strongylodon pulcher*, *Strongylodon zschokkei*, *Strongylodon loheri* and oblong-elliptic in *Strongylodon macrobotrys*. Calyx shape of *Strongylodon juangonzalezii* is cup-shaped which makes it distinct from the rest. Compared with the other species, *Strongylodon juangonzalezii* occurs at lower elevation.

### Key to the species of *Strongylodon* in the Philippines

**Table:** 

1	Inflorescence a raceme, attached on plagiotropic branches; flowers lilac when young, blue when mature; calyx cup-shaped, lilac colored	***Strongylodon juangonzalezii* sp. nov.**
–	Inflorescence a pendulous or drooping raceme; flowers same color all throughout; calyx campanulate, colors various	**2**
2	Brachyblasts cylindric; flowers purplish blue	**3**
–	Brachyblasts warty; flowers variously colored	**4**
3	Flowers axillary; calyx purplish	***Strongylodon caeruleus***
–	Flowers terminal; calyx green	***Strongylodon pulcher***
4	Inflorescence axis > 50 cm long; calyx purplish; flowers bluish green, standard petal 3.5-4.5 cm long, wing petal oblong-elliptic	***Strongylodon macrobotrys***
–	Inflorescence axis < 50 cm long; calyx green to blue; flowers various, standard petal shorter than 3.5 cm, wing petal oblong	**5**
5	Calyx green	**6**
–	Calyx blue	***Strongylodon zschokkei***
6	Inflorescence in compact, subglobose clusters	***Strongylodon elmeri***
–	Inflorescence in loose, drooping clusters	**7**
7	Flowers orange-red, 2–3 in a cluster	***Strongylodon lucidus***
–	Flowers purplish-blue, 5-7 in a cluster	***Strongylodon loheri***

## Supplementary Material

XML Treatment for Strongylodon juangonzalezii
